# Supplementary material for: Determining the scale at which variation in a single gene changes population yields
Source: eLife. 2020 Feb 14;9:e53517. doi: 10.7554/eLife.53517 (PMC7136025; doi:10.7554/eLife.53517)
Supplement: Supplementary file 1. [file elife-53517-supp1.docx]

**Supplementary File 1**

**Table of *emmeans* contrasts, within genotypes from Figure 3-figure supplement 6^α^**

**Model Contrast Trait t-value p-value**

LME EV in 0% irMPK4_(n = 40-44)_ –

EV in 50% irMPK4_(n = 21-22)_ Rosette Diameter -2.786 **0.0268**

Stalk Height -2.644 **0.0438**

Shoot Biomass -3.859 **0.0009**

Water Content -2.130 0.1475

Total Fitness Correlates -3.825 **0.0010**

LME irMPK4 in 100% irMPK4_(n = 31-32)_ –

irMPK4 in 50% irMPK4_(n = 22)_ Rosette Diameter 3.898 **0.0006**

Stalk Height 1.387 0.5091

Shoot Biomass 5.359 **<.0001**

Water Content 3.819 **0.0010**

Total Fitness Correlates 3.794 **0.0011**

^α^extracted from linear-mixed effect (LME) models with significant ANCOVA results
